# Leader autonomy support in the workplace: A meta-analytic review

**DOI:** 10.1007/s11031-018-9698-y

**Published:** 2018-05-17

**Authors:** Gavin R. Slemp, Margaret L. Kern, Kent J. Patrick, Richard M. Ryan

**Affiliations:** 1 Centre for Positive Psychology, Melbourne Graduate School of Education, The University of Melbourne, Level 2, 100 Leicester Street, Parkville, VIC 3010 Australia; 2 Institute for Positive Psychology and Education, Australian Catholic University, Level 9, 33 Berry Street, PO Box 968, North Sydney, NSW 2060 Australia; 3 University of Rochester, Meliora Hall, Rochester, NY 14627 USA

**Keywords:** Autonomy support, Leadership, Motivation, Meta-analysis, Self-determination theory

## Abstract

Leader autonomy support (LAS) refers to a cluster of supervisory behaviors that are theorized to facilitate self-determined motivation in employees, potentially enabling well-being and performance. We report the results of a meta-analysis of perceived LAS in work settings, drawing from a database of 754 correlations across 72 studies (83 unique samples, *N* = 32,870). Results showed LAS correlated strongly and positively with autonomous work motivation, and was unrelated to controlled work motivation. Correlations became increasingly positive with the more internalized forms of work motivation described by self-determination theory. LAS was positively associated with basic needs, well-being, and positive work behaviors, and was negatively associated with distress. Correlations were not moderated by the source of LAS, country of the sample, publication status, or the operationalization of autonomy support. In addition, a meta-analytic path analysis supported motivational processes that underlie LAS and its consequences in workplaces. Overall, our findings lend support for autonomy support as a leadership approach that is consistent with self-determination and optimal functioning in work settings.

**Electronic supplementary material:**

The online version of this article (10.1007/s11031-018-9698-y) contains supplementary material, which is available to authorized users.

## Introduction

Since the industrial revolution, there has been prevalent interest in how leaders can facilitate and sustain motivation and optimal functioning in employees. Arising from various leadership theories over the past century and by building on *self-determination theory* (SDT; Ryan and Deci 2000, 2017), one line of inquiry has suggested that *leader autonomy support* (LAS)—a leadership style that is thought to nurture the inner motivational resources of employees—is well suited to such an objective. LAS is characterized by leaders who take interest in the perspectives of their employees, provide opportunities for choice and input, encourage self-initiation, and avoid the use of external rewards or sanctions to motivate behavior. While this management style has generally been found to yield increased engagement, performance, and well-being (Baard et al. 2004; Deci et al. 2001; Hardré and Reeve 2009), there are some mixed effects across the literature. Further, if LAS is indeed beneficial to employee functioning, clarity is needed as to the theoretical processes involved.

Thus, we appraise the relevant literature by conducting a meta-analytic review of studies that have examined associations between perceived LAS and a variety of employee outcomes, including autonomous and controlled work motivation, basic psychological needs, a range of work-based consequences, and employee well-being. Meta-analysis can provide a quantitative summary of the observed correlations in a literature, identify moderators of those correlations, test theoretical mechanisms, and highlight areas of inquiry that might be pursued in the future. We begin by providing a brief history of LAS in the workplace. We next provide a basic overview of the types of motivation described by SDT and explain how these are thought to relate to LAS in organizations. We review possible moderators of effects, and then explore how LAS relates to basic needs, well-being, and work outcomes, before turning to the meta-analytic review itself.

## Leader autonomy support in work organizations

For many, the motivation and optimal functioning of employees is largely seen to be driven by leader behavior (Gilbert and Kelloway 2014). Thus, there is a growing literature devoted to how leaders can positively affect the motivation and behavior of individual workers (e.g., Barling et al. 2010). Early attention to the topic can be traced to Taylor’s “scientific management”, where subordinate motivation was thought to result from close supervision and controlling practices such as scientifically designed incentive systems (Taylor 1911). Literature later differentiated autocratic from democratic leadership, suggesting that these leadership styles generate different motivational states in followers (Coch and French 1948; Lewin et al. 1939). Autocratic leadership, to the extent that it engenders a climate of fear, was thought to activate fleeting motivational states that persist only while the leader remained physically present. The more consultative democratic style, in contrast, was thought to produce sustained motivated behaviors that persisted even in the absence of the leader. These two contrasting leadership styles set the foundation for later theorizing about the antecedents of intrinsic and extrinsic work motivation (Zaccaro et al. 2008).

More recently, few theories have generated as much scholarly attention on extrinsic and intrinsic forms of motivation as SDT (Ryan and Deci 2000, 2017). SDT is a broad theory of human motivation that concerns individuals’ innate growth tendencies and basic psychological needs, and focuses on the degree to which individual behavior is autonomously motivated or controlled. It has been applied across a variety of research domains, including education, health, sport, parenting, and organizations (e.g., Ng et al. 2012; Reeve 2015; Ryan and Deci 2017; Su and Reeve 2011).

A key focus in the organizational domain has been on the contextual factors that support employee self-determination and basic psychological needs (e.g., Deci et al. 2001; Van den Broeck et al. 2016). Job autonomy, broadly defined as the extent to which individual workers can self-govern how and when they perform the various tasks that make up their job (Hackman and Oldham 1976; Spector 1986), is often identified as a contextual antecedent of self-determination in the workplace (Johns 2006; Ryan and Deci 2017). In addition to the design of jobs (Barrick and Mount 1993), perceived autonomy can stem from interpersonal characteristics, including the leader’s motivating style, which can range from highly supportive to highly controlling (Deci et al. 1989; Gagné et al. 2018; Reeve 2015).

*Leader autonomy support* refers to a cluster of supervisory behaviors that collectively promote a climate of support and understanding within leader-worker relationships (Reeve 2015). An autonomy supportive style generally involves leaders acknowledging worker perspectives, encouraging self-initiation, offering opportunities for choice and input, communicating in an informational rather than a controlling manner, and avoiding the use of rewards or sanctions to motivate behavior (Baard et al. 2004; Hardré and Reeve 2009; Su and Reeve 2011). It is thought to foster more agentic and self-determined pursuits, as recipients perceive themselves to be the regulators of their own actions, fostering a heightened sense that behavior is internally directed rather than externally controlled (Deci et al. 2017). In contrast, a controlling leadership style involves leaders imposing external constraints on behavior with the intention of compelling individuals to produce specific outcomes (Ryan et al. 1983). A controlling style is often interpreted as prescriptive, inflexible, and rigid, pressuring the employee to think, feel, or behave in particular ways (Ryan and Deci 2017). Deviations from leader demands are often met with corrective or other punitive actions intended to restore behavior back to its desired course. Hence, the style signals to employees that the leader is the initiator of action, shifting the perceived cause of one’s behavior to an external source (Deci et al. 1989; Deci and Ryan 1987).

Interest in organizational applications of LAS first emerged in field studies in corporate settings. For instance, Deci et al. (1989) observed that when employees in a Fortune 500 firm perceived their direct manager as supporting their autonomy, their satisfaction with their supervisors, job satisfaction, and trust in the senior organizational leaders tended to be elevated. Since then, research on autonomy support in leadership has grown substantially, with autonomy supportive practices shown to predict positive work behavior (e.g., proactive and prosocial work behavior; Gagné 2003; Slemp 2017; Slemp et al. 2015), as well as employee well-being and work engagement (e.g., Deci et al. 2001; Moreau and Mageau 2012; Schultz et al. 2015; Williams et al. 2014). Other studies have explored work-based correlates of LAS, including job attitudes (e.g., Collie et al. 2016), performance (e.g., Baard et al. 2004; Braun et al. 2012), and turnover intentions (e.g., Gillet et al. 2013; Liu et al. 2011). But perhaps the most prevalent area of inquiry has explored how LAS relates to motivational processes, including basic psychological needs at work (e.g., Van den Broeck et al. 2016), and various forms of work motivation, to which we turn next.

## Leader autonomy support and the autonomous motivation of work behavior

**Fig. 1:**
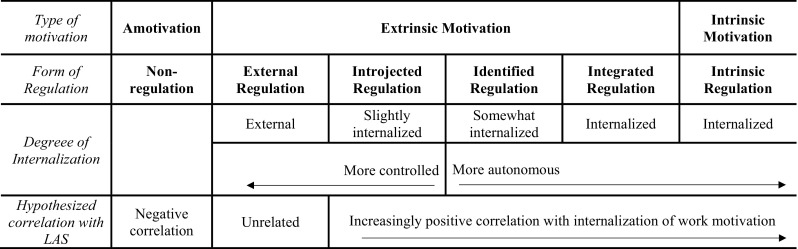
The organismic integration continuum showing the degree of internalization and autonomy associated with each type of motivation, and expected relations with LAS. Figure adapted from Ryan and Deci (2000)

Central to SDT is the distinction between forms of autonomous motivation, controlled motivation, and amotivation (see Fig. 1; Gagné et al. 2018; Howard et al. 2016; Ryan and Deci 2000). Autonomous motivation encompasses behaviors emanating from within the self, involving a sense of volition and choice. *Intrinsic motivation* is a prototype of autonomous motivation, and involves engaging in an action because it is interesting or enjoyable. Other autonomous forms of motivation are *identified* and *integrated regulation*. When acting through *identified regulation*, the individual recognizes the value of a behavior and is volitionally motivated to enact it. *Integrated regulation* occurs when identified values fit together and are congruent, such that the person can be wholeheartedly engaged. In contrast, controlled motivation involves behaviors that are performed due to causes perceived to be external to the self, and thus the volitional component of behavior is either partially or completely absent. A highly controlled form of behavior is represented by *external regulation*, in which one is motivated by externally administered contingencies such as tangible rewards or avoiding punishment. Slightly less controlled is *introjected regulation*, where an individual begins to internalize and value the external contingencies sought in external regulation. When introjected, the pressures on the individual are internal, and self-esteem is contingent on one’s behaviors. Beyond these categories of autonomous and controlled motivation is *amotivation*, which involves no desire to enact behavior. Amotivation has two broad sources or subtypes—amotivation due to lack of perceived control or efficacy; and amotivation due to lack of value or concern, both of which can be problematic in the workplace when the behavior needs to be enacted.

An important part of this SDT framework is the process of *internalization*, which refers to a natural tendency for people to transform controlled motivation into more autonomous forms of motivation (Ryan and Deci 2000). Support for basic psychological needs for autonomy, competence and relatedness is seen as essential to fuller internalization, and among these supports, autonomy support is particularly central (Ryan and Deci 2017). Autonomy support includes such elements as taking the actor’s internal frame of reference (empathy), provisions of rationale and choice, and minimal use of controls to motivate. Within an autonomy supportive context, individuals are encouraged to value and assume responsibility for behaviors or goals (Deci et al. 1994). Thus, LAS should theoretically be more positively and strongly correlated with the more internalized motivational processes described by SDT (Gagné et al. 2015; Ryan and Deci 2017), as illustrated in Fig. 1.

Whereas LAS is likely to show positive and progressively stronger correlations with the more internalized motivational processes towards the right of Fig. 1, we expect that for externally regulated behavior, given its lack of internalization, correlations will generally be unrelated to LAS (Gagné et al. 2015; Ryan and Deci 2017). This is because LAS is more likely to exert its positive effects on individuals’ more autonomous and intrinsic forms of motivation, which have typically been uncorrelated with external regulation (Ryan and Deci 2000, 2017). In external regulation, one’s motivation is focused on factors external to the self, such as pay or incentives, whereas an autonomy supportive manager would be supporting a sense of ownership, interest and value, reflected in identified and intrinsic motives. Depicted as well in Fig. 1 is our expectation that LAS will, in contrast, show a significant negative correlation with amotivation. Since autonomy support fosters an environment where individuals experience willingness and choice, people should be more motivated to engage in their work, and amotivation is more likely to be absent. Thus, unlike external regulation which has typically been uncorrelated with highly autonomous motives, amotivation has typically been negatively associated with them, and we expect a similar a pattern in how LAS relates to these motivational types.

Notably, although many studies support these general patterns of relations (e.g., Collie et al. 2016; Gagné et al. 2015), others do not. For instance, correlations between LAS and introjected regulation range from strongly positive (*r* = .42; Nie et al. 2015) to moderately negative (*r* = − .18; Krieger and Sheldon 2015). The strong correlation of LAS and introjection in the Nie et al. study was accompanied by even stronger positive relations of LAS to intrinsic and identified motivations, as well as a strong negative relation with external regulation (*r* = − .38). It is possible therefore that the sample of Chinese teachers in this study were not only more autonomously motivated by higher autonomy support, but also felt greater internal pressure “not to disappoint”. Mixed findings can also be observed in other studies (e.g., Allen and Bartle 2014). Correlations between LAS and intrinsic motivation range from relatively small (*r* = .15; Roche and Haar 2013) to large (*r* = .56, Olafsen et al. 2015), with similar ranges for identified regulation. Correlations with external regulation are similarly varied, with some studies showing positive relations (e.g., Jones 2002; Oostlander et al. 2014) and others showing negative relations (e.g., Nie et al. 2015). By statistically aggregating previous findings through meta-analysis, we hope to provide clarification about the strength of the associations between LAS and the full range of motivational processes discussed in SDT.

### Hypothesis 1

LAS will show a pattern of stronger and increasingly positive correlations with more internalized forms of work motivation.

## Moderators of leader autonomy support and motivational processes

The heterogeneity observed in prior studies between LAS and motivational processes, including internalization of work motivation or basic need satisfaction, also point to a need to understand study and participant characteristics that impact these associations. Meta-analysis is particularly well-suited to testing moderating factors (Rosenthal and DiMatteo 2001). While any number of moderators could be explored, depending on appropriate data being available, we make two theoretically derived predictions concerning possible factors that moderate correlations between LAS and its motivation-based correlates.

First, there is evidence to suggest that the effects of leader behaviors on employees may be affected by the physical and/or psychological distance between the leader and employee (Antonakis and Atwater 2002). The associations between transformational leadership and follower behavior, for example, has been found to be attenuated by increased social (e.g., Cole et al. 2009) or physical (e.g., Howell and Hall-Merenda 1999; Humphreys 2002) distance. Based on the available studies, we broadly define distance in terms of the supervisor and employee hierarchy within the organization, ranging from proximal (i.e., immediate supervisor) to more distal sources (e.g., senior organizational leaders). It is likely that the salience of leader behavior is heightened within proximal relationships because it is more commonly observed, and thus observed correlations may be stronger in these situations.

### Hypothesis 2

Autonomy support from proximal leaders will show stronger correlations with motivation and basic needs than autonomy support from more distal leaders.

Second, prior research has suggested that perceived autonomy predicts well-being across cultures (e.g., Chirkov et al. 2003; Ryan 1995), suggesting that the need for LAS may be culturally universal. But studies have not directly compared the impact of LAS on psychological needs and motivation in employees across cultures. While culture can be classified in a variety of ways, a basic classification that has been used in the literature—and which we use here—is between individualist and collectivist cultures (Oyserman 2017).

### Hypothesis 3

Correlations between LAS and motivation will not differ as a function of whether the sample is drawn from an individualist or collectivist country.

While the moderators previously reviewed are theoretically driven, there are a variety of study-related factors that can also yield differences in the observed effects. Two factors that can bias results of meta-analyses are publication bias and imperfect construct validity in measurement instruments (Schmidt and Hunter 2015). If publication bias is present in a literature, the likely upshot is an upward bias in mean effect sizes and a downward bias in the variability across effect sizes due to “missing” small effect-size studies (Schmidt and Hunter 2015; Schmidt and Oh 2016). Hence, publication bias is known to falsely inflate the results of research literatures. To explore this possibility in the present meta-analysis, we test publication status as a possible moderator of observed effects, and we also explore evidence of small study bias (Borenstein et al. 2009).

In addition, several instruments have been used to measure perceived leader autonomy support in the literature, including the short- and long-form of the Work Climate Questionnaire (Baard et al. 2004), the Perceived Autonomy Support Scale for Employees (Moreau and Mageau 2012), and the Work Climate Scale (Deci et al. 1989), among other idiosyncratic measures (e.g., Lynch et al. 2005). Because correlations are attenuated by imperfect construct validity in measurement instruments (Schmidt and Hunter 2015), to the extent that any of these measures are less construct-valid than others, their yielded effects will likely be systematically biased downwards. Thus, the operationalization of autonomy support is important to consider as a possible moderator of observed effects and we explore this possibility in the present meta-analysis.

We had no a priori hypotheses for either publication bias or the operationalization of autonomy support, and thus present these moderators as exploratory results.

## Relations of leader autonomy support to basic needs, well-being, and work outcomes

SDT posits that individuals experience optimal psychological functioning to the extent that three basic psychological needs for autonomy, competence, and relatedness are satisfied (Deci and Ryan 2000). *Autonomy* involves a sense of choice and freedom in one’s behavior, and entails the perception that one’s behavior is a function of one’s own interests and values rather than being controlled by forces or pressures external to the self. *Competence* involves feelings of mastery, attaining desired outcomes, and succeeding at challenging tasks. *Relatedness* involves an ability to develop meaningful relationships and connection with others.

Leaders play an important role in establishing and maintaining a social context that allows employees to feel free to pursue experiences that satisfy these three needs. SDT argues that, because autonomy supportive leaders attempt to understand the perspective of their employees, they are more likely to facilitate employee experiences of autonomy, competence, and relatedness (Ryan and Deci 2017). When operating within an autonomy supportive workplace, employees can engage in more self-directed behaviors, more freely address obstacles and challenges, and feel more support and connection (Deci et al. 2001). LAS may therefore be a potent social-contextual motivational precursor to fulfilling these three psychological needs (Baard et al. 2004; Gagné 2003). This is consistent with recent meta-analytic findings (Van den Broeck et al. 2016) that found positive associations with autonomy (ρ = .65; *k* = 13), competence (ρ = .38; *k* = 13), and relatedness (ρ = .39; *k* = 14).

Because the satisfaction of autonomy, competence, and relatedness tends to catalyze intrinsic enjoyment and/or value and satisfaction in activities themselves, need satisfaction is understood to be essential for the internalization of motivation. As noted by Gagné (2003), “People are more likely to be intrinsically motivated … when they can freely choose to pursue the activity (autonomy), when they master the activity (competence), and when they feel connected and supported by important people, such as a manager, a parent, a teacher, or team-mates (relatedness)” (p. 202). On this basis, prior work tends to position basic need satisfaction as a conceptual and empirical antecedent to autonomous work motivation (e.g., Collie et al. 2016; De Cooman et al. 2013).

SDT also predicts that need satisfaction results in individual well-being (Olafsen 2017). Numerous theories and definitions of psychological well-being exist, including ideas stemming from both hedonic (e.g., subjective well-being) and eudaimonic (e.g., meaning/purpose) traditions (Deci and Ryan 2008; Ryan and Deci 2001). While there is some evidence that LAS predicts both forms of well-being, it has not yet been established whether it relates more strongly to hedonic indicators, or feelings and experiences associated with eudaimonia, as few studies have examined both simultaneously. This is important because an understanding of the environmental conditions that support different types of well-being will allow for more targeted interventions (Kern et al. 2015).

Beyond well-being, the need satisfying experiences and internalization of motivation produced by LAS may also impact a variety of workplace outcome variables, including work performance, job satisfaction, work engagement, and various work-related behaviors (e.g., proactive or prosocial behavior) (Deci et al. 2017). When employees perceive that they are free to perform their work in their own way within an autonomy supportive context, they may be more likely to find that work engaging, possess more favorable evaluations of the job (job satisfaction), and proactively engage with their environment and others with whom they work (proactive and prosocial behavior, and work performance). Overall, the empirical data is largely consistent with these premises, showing positive relations between LAS and work engagement (e.g., Deci et al. 2001; Van Schie et al. 2015), positive job attitudes (e.g., organizational commitment; Chang et al. 2015; Collie et al. 2016) prosocial and proactive behaviors (e.g., Gagné 2003; Güntert 2015; Slemp 2017; Slemp et al. 2015), and performance (e.g., Baard et al. 2004; Braun et al. 2012).

**Fig. 2:**
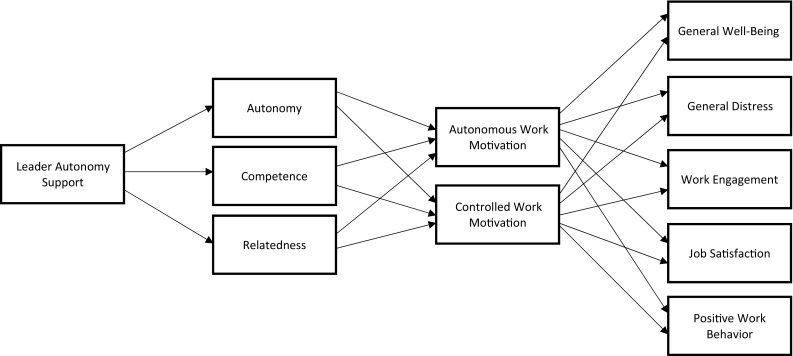
Path diagram of the hypothesized associations amongst the study variables

To summarize, Deci et al. (2017) suggested a model of work motivation that posits how the SDT constructs relate with each other within the organizational literature. Figure 2 adapts their model specifically to focus on LAS. From this perspective, autonomy support is a social-contextual antecedent that fosters basic need satisfaction, which in turn leads to the internalization of work motivation, which leads to enhanced employee health (e.g., employee well-being and lower distress) and work outcomes (e.g., performance, job satisfaction, work engagement). While some studies have tested various parts of Fig. 2, no study to our knowledge has simultaneously tested the full model. Thus, our specific predictions regarding this proposed model are:

### Hypothesis 4

LAS will predict higher levels of autonomy, competence and relatedness;

### Hypothesis 5

Autonomy, competence, and relatedness will in turn predict higher levels of autonomous versus controlled work motivation;

### Hypothesis 6

Autonomous motivation, relative to controlled motivation, will in turn predict enhanced employee health and positive work outcomes.

## The present study

Although several studies have examined correlates of LAS in workplaces, no attempt has yet been made to statistically aggregate the findings in this literature. Accordingly, the exact magnitude of its relationship with important variables, including the internalized work motivation processes described by SDT, as well as well-being, behavior, and other work-based outcomes have not yet been established. Meta-analysis can help address this need because it makes possible the systematic combination and statistical aggregation of single studies to obtain more stable estimates of overall associations, as well as moderators of those associations (Schmidt and Hunter 2015).

In conducting our meta-analysis, we had three objectives. First, we aimed to identify and quantify the relations between perceived LAS and a range of variables that have been tested in the organizational literature, presenting broad descriptive evidence of its most common correlate variables with estimates of the size of the corrected correlations. As illustrated in Fig. 2, studies have primarily treated autonomy support as a predictor. Because few studies have explored potential antecedents of LAS, particularly from the leaders’ perspective, including age, gender, tenure, organizational seniority, or relevant personality characteristics (e.g., agreeableness), we primarily focus on the theoretical outcomes associated with LAS.

Second, we aimed to test potential moderators of the observed correlations. We consider the distance between the employee and supervisor and compare individualist and collectivist cultures as potential influences. We also explore publication bias and how autonomy support was operationalized as potential study-level factors. Identification of moderators can help explain prior inconsistent findings in the literature, and may also help guide future research efforts.

Third, we aimed to test the hypothesized pathway model summarized in Fig. 2. Integrative reviews make it possible to bridge across samples, even when no one primary study has investigated all of the variables of interest (Becker 2009; Schmidt 2010; Viswesvaran and Ones 1995). Because the overall correlations connect the studies together, pathways can be tested, and theoretical predictions can be explored. This technique is important because it allows us to go beyond the individual meta-analysis results and assess the relative impact of the key theoretical mechanisms intended to explain the autonomy support to work motivation and work outcome associations proposed by Deci et al. (2017).

## Method

### Literature search strategy

We searched six electronic databases for relevant articles published through October 2016: PsycINFO, Web of Science, ProQuest Dissertations and Theses Global, ERIC, Academic Search Complete, and Business Source Complete. We used two sets of search terms:

- **Set 1, autonomy support**: “autonom* support”, “support for autonomy”, “needs support”
- **Set 2, workplace context**: “leader*”, “workplace”, “organi*ation”, “employee”, “staff”, “work-unit”, “company”, “business”, “work-based”, “occupation*”, “job”, “vocation*”

Searches were initially conducted using set 1. Set 2 was added to refine the search when the individual databases’ hits exceeded 2000, which happened in one instance. No date, geographical, or cultural restrictions were imposed on the searches, but articles were limited to English. We then snowballed the reference lists of key papers for relevant studies. Next, we searched Google Scholar, PsycINFO, and Web of Science for articles that had cited key papers associated with a work-based perceived autonomy support scale.^1^ Finally, we posted calls for unpublished studies by contacting notable SDT researchers.

The search process identified 4607 potential articles through databases, and another 720 through reference list snowballing. These sources were initially screened for inclusion using the titles and abstracts. This initial screening process led to the exclusion of 4611 studies due to duplication, irrelevancy, or clear failure to meet the inclusion criteria (see below). The resulting set of 716 articles was reduced to 112 based on eligibility criteria. An additional 40 were removed for failing to provide enough information to extract a correlation and, where possible, requests for data from the corresponding author were either not answered or unavailable. Finally, eligible sources were screened using the procedure described by Wood (2008) to eliminate bias created by duplicate studies. In all, a database including a total of 72 sources (50 published), reporting data from 83 unique samples and 32,870 participants were included in the current meta-analysis (see Appendix 1 in Supplementary Material for systematic search flow diagram).

### Inclusion criteria

Studies were included in the meta-analysis if they satisfied five criteria: (a) The study included non-clinical adult participants examined within an organizational setting. Studies that used psychiatric or other healthcare patients or community samples were excluded. Student samples were only included if they were examined within an organizational setting (e.g., MBA interns studied on placement). (b) The study reported a correlation coefficient between autonomy support and at least one of the following outcome variables: work motivation (autonomous or controlled), the satisfaction of one or more psychological needs (autonomy, competence, or relatedness), employee well-being, work engagement, psychological distress, job attitudes, turnover intentions, prosocial behavior (e.g., organizational citizenship behaviors; knowledge sharing), proactive behavior (e.g., job crafting; feedback seeking), mindfulness, work performance, autonomy orientation, or demographic variables (age, gender, and organizational tenure). (c) Studies needed to focus on the workers’ perspective and thus examine *perceived* leader autonomy support. Studies that focused solely on the leaders’ perspective of their own autonomy supportive supervision style were excluded. (d) The focus of the study needed to be LAS as an important part of the social work context. Studies that focused exclusively on job autonomy as a feature of work design were excluded. (e) Correlations and reliability coefficients needed to be reported at the individual level, as our primary interest in this study was in correlates of LAS for individual employees. Studies reporting only team or business unit-level data were excluded.

### Coding procedure

The initial set of studies, produced by database searches and reference list snowballing, were coded by two of the authors using a systematic coding sheet (see Appendix 2 in Supplementary Material). An accuracy check revealed 94.8% agreement across all coding categories. Disagreements were resolved via discussion. Given the high interrater reliability, any study retrieved after the initial set was established was coded solely by the first author. Studies were coded on 12 categories: (a) sample size; (b) size of the correlation between autonomy support and the correlate variables; (c) the reliability of the autonomy support scores, (d) the scale used to measure autonomy support; (e) the reliability of the correlate scores; (f) the name of the correlate variables; (g) the locus of autonomy support (proximal vs. distal); (h) the publication status of the study (published versus unpublished); (i) time lag between the measurement of autonomy support and correlate variables (if any); (j) year of publication; (k) occupation of the participants; and (l) country where the study was conducted. Drawing on the work of Hofstede (2001), each country was further classified as individualist (e.g., United States) or collectivist (e.g., China).

### Data transformations

Our coding process involved two transformations of the data. First, we used the formula provided by Schmidt and Hunter (2015) to aggregate within study correlations when the original source only presented correlations involving facets of autonomy support or facets of the correlate variables. For example, some studies (e.g., Collie et al. 2016; Gagné et al. 2015) provided separate correlations for facets of autonomous motivation (e.g., intrinsic motivation, identified regulation) and controlled motivation (introjected regulation, external regulation) using the Multidimensional Work Motivation Scale (Gagné et al. 2015). The correlations between these facet variables were used to arrive at composite correlations between perceived LAS and both autonomous and controlled forms of work motivation. We also calculated separate meta-analytic estimates for the more specific motivational facets. Where necessary, similar procedures were used to calculate composite correlations between perceived LAS and intrinsic need satisfaction (total), work engagement, organizational commitment, proactive behavior, and prosocial behavior. Moreover, because the facets of autonomy support or its criterion constructs were generally not orthogonal, Mosier (1943) reliabilities for composite variables were calculated when possible. The calculation of the Mosier reliabilities requires the correlations between facets within a composite. In the few instances where these data were unavailable we entered the mean of the reliabilities across the facets as a proxy.

Second, studies have investigated correlations between perceived LAS and a range of correlate variables. Meta-analysis requires the grouping of similar variables together, which we used to establish correlate categories. For example, Atkins et al. (2015) measured well-being using two measures: positive affect and life satisfaction. The correlations between these variables were statistically aggregated to arrive at an overall estimate of the correlation between autonomy support and hedonic well-being, which was then entered into the meta-analysis (see Appendix 3 in Supplementary Material for a full list of the measured variables used to construct correlate categories).

### Meta-analytic procedure

We used the Schmidt and Hunter (2015) psychometric meta-analytic method in conducting the analyses, using the Schmidt and Le (2014) meta-analysis software. First, we calculated a sample size weighted mean correlation between LAS and each correlate variable. Second, because most studies reported reliability data (Total = 83% for *R*_*xx*_, 78% for *R*_*yy*_), correlations were individually corrected for measurement error in both the predictor and the correlate variable. For those studies that did not report reliabilities, we used the mean of the reliabilities reported in the included studies for that variable. Finally, a disattenuated true correlation was estimated between LAS and each correlate variable.

The Schmidt and Hunter (2015) approach to meta-analysis is based on the random effects model, which allows parameters to vary across studies and provides an estimate of the variance in effect sizes. The adoption of random effects models is supported by studies showing they lead to more accurate population effect size estimates that are generalizable beyond the database included in the analysis (Field 2003; Hunter and Schmidt 2000; Kisamore and Brannick 2008; Schmidt 2010). A 95% confidence interval (CI) was constructed around each correlation. When the CIs encompassed 0, which suggests a possible true correlation of 0, we concluded that the relation between the two constructs was of no substantive significance. As Cohen (1988) effect size benchmarks bear little resemblance to effect size distributions in applied psychology (Bosco et al. 2015), we instead used Bosco et al.’s (2015) job attitudes-people attitudes distribution to gauge the strength of our findings. Using 33rd and 67th percentiles of this distribution, correlations of up to .18 were deemed small, .19 to .35 were deemed moderate, and .36 and above were deemed large.

We calculated the total variance in the correlations, as well as that attributable to the study artifacts of sampling and measurement error. Homogeneity was assessed with the 75% rule of thumb (Schmidt and Hunter 2015), which suggests that if 75% or more of the variance is due to the corrected known artifacts including sampling and measurement error, the remaining 25% is likely due to artifacts for which no correction has been made and the effect sizes are homogenous. Thus, where this variance was less than 75% and where sufficient studies were available, we explored theoretically relevant or study-related moderator variables. Moderator analyses were run by conducting a series of meta-analyses carried out separately across the different levels of the moderator. A variable was deemed to be a moderator if the CIs of the separated effect sizes did not overlap (Borenstein et al. 2009; Schmidt and Hunter 2015). The width of the credibility interval (CV) is another useful way to suggest the presence of moderators (Whitener 1990), and we include it to supplement the percentage of variation in observed correlations explained by study artifacts.

Meta-analytic estimates were computed whenever at least three studies reported a relation between LAS and the correlate variable. To minimize the effect of common method variance in our data, when studies included both cross-sectional and lagged correlations, we only included the lagged correlations consistent with the causal direction implied by our hypothesized path model, showing autonomy support as the predictor (Fig. 2). We summarize the meta-analytic findings with eight pieces of information: (a) *k* = number of studies used to calculate meta-analytic estimates, (b) *N* = sample size used to calculate each estimate, (c) *r*_*obs*_ = sample size weighted observed correlation, (d) ρ = estimate of the true score correlation, (e) *SD*_ρ_ = standard deviation of the true score correlations, (f) 95% CI = 95% confidence interval, (g) 80% CV = 80% credibility interval, and (h) % artifacts = percentage of variation in observed correlations attributable to the study artifacts of sampling and measurement error.

### Meta-analytic path analysis

To evaluate the hypothesized model in Fig. 2, we subjected the meta-analytically derived correlations to path analysis. Because attenuation caused by measurement error can produce biased path coefficients (Hunter and Gerbing 1983; Schmidt et al. 1986), we used three approaches to ensure the correlations in the matrix were corrected for both sampling and measurement error.

First, correlations generated in this study were used to fill all possible cells in the matrix. Second, results from previously published meta-analyses were used to fill possible remaining cells. This primarily included the results of Van den Broeck et al. (2016) for their meta-analytic estimates relating to basic psychological needs in the workplace. We also used these estimates if their associated samples were larger than those in our study database. Finally, some missing cells remained in the matrix, relating to correlates of controlled motivation and work engagement. We sourced an additional set of studies to compute these estimates. To do this, we prospectively searched the citing studies of the Multidimensional Work Motivation Scale (MWMS; Gagne et al. 2015) and the Utrecht Work Engagement Scale (UWES; Schaufeli et al. 2006), which were the most commonly used measures for these variables from the present study. Due to the substantial number of citing studies of the UWES, we refined the search by using key words for the missing correlate variables in the matrix and limited the search to 2014–2017. This process yielded a total of *k* = 20 further studies (n = 9822) to compute estimates for controlled motivation, and *k* = 19 further studies (n = 13,738) to compute estimates for work engagement.

Four fit indices were used to test the fit of the model: the comparative fit index (CFI), the Tucker Lewis Index (TLI), the standardized root mean square residual (SRMR), and the root mean square error of approximation (RMSEA). Recommendations of Hu and Bentler (1999) suggest relatively good model fit is indicated by values exceeding 0.90 for the TLI and above .95 for the CFI, and values less than .06 for the SRMR and .08 for the RMSEA.

## Results

### Relations of leader autonomy support to employee outcomes

Table 1 summarizes meta-analytic findings for associations between perceived LAS and various outcomes considered in the organizational literature. We split our reporting of these results into those outcomes coming under: (a) motivational and psychological processes; (b) employee well-being and distress; (c) work-based outcomes; and (d) employee demographics.

**Table 1:** Separate meta-analytic estimates of the relations between leader autonomy support and its relevant correlate variables

Correlate	*k*	*N*	*r*_*obs*_	ρ	*SD*_ρ_	95% CI		80% CV		% Artifacts
						Lower	Upper	10%	90%	
*Motivational and psychological processes*										
Autonomous motivation^a,b^	31	16,597	.34	.38	.08	.35	.42	.28	.48	24
Intrinsic motivation	22	13,654	.34	.38	.07	.35	.42	.29	.47	25
Identified regulation	12	9676	.26	.31	.05	.27	.34	.24	.37	38
Controlled Motivation^b^	16	11,178	.00	.00	.18	− .09	.09	− .22	.23	6
Introjected regulation	12	9672	− .03	− .04	.20	− .16	.08	− .30	.22	4
External regulation	12	9678	.00	.00	.12	− .07	.07	− .16	.16	10
Amotivation	7	2220	− .28	− .31	.11	− .40	− .22	− .45	− .17	23
Autonomy Orientation	13	4441	.19	.23	.12	.16	.31	.09	.38	23
Basic needs total	32	13,343	.48	.55	.10	.51	.59	.42	.68	16
Need for autonomy	25	10,836	.46	.57	.10	.53	.62	.45	.70	19
Need for competence	27	11,636	.34	.42	.18	.35	.48	.19	.64	8
Need for relatedness	26	11,597	.38	.46	.09	.42	.50	.35	.58	24
Mindfulness	5	1550	.15	.16	.11	.06	.27	.03	.30	24
*Employee well-being and distress*										
General well-being^c^	26	12,876	.39	.46	.14	.41	.52	.29	.64	10
Hedonic well-being	18	10,342	.39	.46	.14	.39	.53	.28	.64	8
Eudaimonic well-being	9	3640	.33	.40	.17	.29	.52	.19	.62	9
General distress	25	11,423	− .29	− .33	.10	− .38	− .29	− .47	− .20	18
Burnout	8	2213	− .23	− .27	.20	− .41	− .14	− .52	− .03	11
Work stress	10	3099	− .22	− .25	.13	− .34	− .16	− .42	− .08	18
*Work outcomes*										
Job satisfaction	22	7685	.49	.56	.12	.50	.61	.40	.71	13
Organizational commitment^d^	8	2940	.48	.52	.06	.47	.58	.45	.60	35
Affective commitment	5	2199	.50	.55	.00	.52	.58	.55	.55	100
Normative commitment	4	698	.28	.32	.00	.28	.37	.32	.32	100
Work engagement	18	6397	.29	.33	.05	.30	.37	.26	.40	51
Work performance	14	3259	.22	.25	.15	.17	.34	.06	.44	19
Turnover intentions	9	3057	− .36	− .40	.13	− .49	− .31	− .56	− .24	15
Prosocial behavior	13	4815	.23	.26	.08	.20	.31	.15	.36	31
Proactive behavior	4	1146	.36	.39	.03	.33	.46	.36	.43	82
*Demographics*										
Age	16	5033	.02	.02	.06	− .03	.06	− .06	.09	55
Gender (male = 0, female = 1)	14	6418	− .03	− .03	.05	− .07	.00	− .10	.03	51
Organizational tenure	10	3133	.01	.01	.09	− .05	.08	− .10	.12	33

*k* number of studies in the analysis, *N* combined number of participants, *r*_*obs*_ sample size weighted mean observed correlation; ρ estimate of the true score correlation, *SD*_ρ_ standard deviation of estimated true score correlation, CI confidence interval, CV credibility interval, *% artifacts* percentage of variation in the observed correlations attributable to sampling and measurement error

^a^A meta-analytic estimate is absent for integrated regulation due to *k* < 3 studies.

^b^Correlations between leader autonomy support and autonomous and controlled motivation included composite correlations made up of intrinsic and identified regulation (autonomous motivation) and introjected and external regulation (controlled motivation)

^c^General well-being includes composite correlations made up of both hedonic and eudaimonic domains

^d^An estimate for continuance commitment is absent due to *k* < 3 studies

#### Motivational and psychological processes: internalization, basic needs, and mindfulness

As shown in Table 1, LAS and autonomous motivation were positively correlated (*k* = 31, *N* = 16,597, ρ = .38 [CI .35, .42]). A near zero correlation was found with controlled motivation (*k* = 16, *N* = 11,178, ρ = .002 [CI − .09, .09]), with a lower bound confidence interval encompassing 0. At the facet level, LAS was most strongly related to intrinsic motivation (*k* = 22, *N* = 13,654, ρ = .38 [CI .35, .42]), followed by identified regulation (*k* = 12, *N* = 9676, ρ = .31 [CI .27, .34]). LAS exhibited a near zero association with introjected regulation (*k* = 12, *N* = 9672, ρ = − .04 [CI − .16, .08]) and was unrelated to external regulation (*k* = 12, *N* = 9678, ρ = .00 [CI − .07, .07]). As expected, LAS was moderately negatively correlated with amotivation (*k* = 7, *N* = 2220, ρ = − .31 [CI − .40, − .22]).^2^

For basic psychological needs, LAS was most strongly associated with autonomy (*k* = 25, *N* = 10,836, ρ = .57 [CI .53, .62]). Strong positive associations were also found for competence (*k* = 27, *N* = 11,636, ρ = .42 [CI .35, .48]) and relatedness (*k* = 26, *N* = 11,597, ρ = .46 [CI .42, .50]) needs. Finally, LAS showed a weak positive correlation with employee mindfulness (*k* = 5, *N* = 1550, ρ = .16 [CI .06, .27]).

#### Employee well-being and distress

As shown in Table 1, LAS was strongly correlated with general well-being (*k* = 26, *N* = 12,876, ρ = .46 [CI .41, .52]), with estimates consistent for hedonic (*k* = 18, *N* = 10,342, ρ = .46 [CI .39, .53]) and eudaimonic (*k* = 9, *N* = 3640, ρ = .40 [CI .29, .52]) indicators. LAS was moderately negatively correlated with employee psychological distress (*k* = 25, *N* = 11,423, ρ = − .33 [CI − .38, − .29]). For specific components of distress, LAS showed a moderate negative correlation with both employee burnout (*k* = 8, *N* = 2213, ρ = − .27 [CI − .41, − .14]) and work stress (*k* = 10, *N* = 3099, ρ = − .25 [CI − .34, − .16]).

#### Work-based outcomes

Table 1 shows meta-analytic associations between LAS and employee work-based correlates, including job attitudes, work engagement, work performance, turnover intentions, and work behavior (proactive and prosocial behavior). Job attitudes can broadly be considered as the psychological tendency to evaluate one’s job, or characteristic thereof, with some degree of favor or disfavor (Judge and Kammerer-Mueller 2012). For specific job attitudes, we found sufficient information to compute estimates for job satisfaction and organizational commitment. LAS showed a strong positive correlation with job satisfaction (*k* = 22, *N* = 7685, ρ = .56 [CI .50, .61]) and organizational commitment (*k* = 8, *N* = 2940, ρ = .52 [CI .47, .58]). Considering facets of commitment, LAS showed a stronger correlation with affective (*k* = 5, *N* = 2199, ρ = .55 [CI .52, .58]) than normative commitment (*k* = 4, *N* = 698, ρ = .32 [CI .28, .37]). Continuance commitment is absent due to insufficient studies (*k* < 3).

LAS was moderately associated with employee work performance (*k* = 14, *N* = 3259, ρ = .25 [CI .17, .34]), although the width of the credibility interval (see Table 1) suggests that this association is moderated by other factors. We ran a supplemental analysis to test whether the relation to performance was moderated by the nature of the performance measure, which we coded as self-report or other (e.g., peer ratings, supervisor ratings, or objective performance). The relation between LAS and self-report performance was higher (*k* = 7, *N* = 1626, ρ = .35 [CI .26, .44]) than it was with other types of performance measures (*k* = 7, *N* = 1633, ρ = .15 [CI .05, .26]) suggesting the relation is moderated by the nature of the performance measure.

LAS was moderately positively correlated with work engagement (*k* = 18, *N* = 6397, ρ = .33 [CI .30, .37]), and strongly negatively correlated with turnover intentions (*k* = 9, *N* = 3057, ρ = − .40 [CI − .49, − .31]). It showed a strong positive association with proactive work behavior (*k* = 4, *N* = 1146, ρ = .39 [CI .33, .46]) and a moderate positive association with prosocial work behavior (*k* = 13, *N* = 4815, ρ = .26 [CI .20, .31]).

#### Employee demographics

Finally, Table 1 includes available employee demographic variables (age, gender, and organizational tenure), which we extracted where available. LAS showed no substantive association with demographic variables, with confidence intervals all encompassing 0.

### Moderator analyses

Our second aim was to examine possible moderators of the observed correlations where heterogeneity was present. First, we considered the source of autonomy support as a moderator (Hypotheses 2), based on whether it was coming from a direct supervisor (coded as *proximal* autonomy support) or a more distant source (e.g., senior leader, general environment; coded as *distal* autonomy support). Despite most studies failing to report separate associations between the different types of autonomy support and its correlate variables, we were able to extract sufficient information to explore this moderator in relation to basic needs. Both the Work Climate Scale (Deci et al. 1989) and the Lynch et al. (2005) measures include subscales to explore autonomy support from these different sources. Similarly, the Perceived Autonomy Support Scale for Employees (Moreau and Mageau 2012) has leader as well as colleague autonomy support built into it, but few studies reported separated results for this. Generally, studies that used the Work Climate Questionnaire (Baard et al. 2004) were coded as assessing proximal autonomy support, since the items generally refer to the employee’s direct supervisor, though there were instances where these scales were adapted to assess autonomy support from more distal sources (e.g., Liu and Fu 2011). Results showed no evidence of moderation effects, with proximal and distal autonomy support showing similar associations with the need for autonomy (*proximal: k* = 22, *N* = 9894, ρ = .57 [CI .52, .61]; *distal: k* = 3, *N* = 942, ρ = .59 [CI .37, .81]), competence (*proximal: k* = 22, *N* = 9384, ρ = .42 [CI .36, .48]; *distal: k* = 6, *N* = 1765, ρ = .46 [CI .28, .63]), and relatedness (*proximal: k* = 20, *N* = 9179, ρ = .38 [CI .33, .43]; *distal: k* = 6, *N* = 1765, ρ = .39 [CI .28, .50]).

Second, to explore the possibility that cultures do not differ in the effects of LAS on integrative processes such as internalization and basic need satisfaction (Hypothesis 3), we calculated and compared separate estimates based on country (individualist versus collectivist). As above, we explored this in relation to basic needs, and found no evidence of moderation, with both cultures yielding similar results for autonomy (*individualist: k* = 19, *N* = 9408, ρ = .57 [CI .52, .62]; *collectivist: k* = 6, *N* = 1428, ρ = .54 [CI .45, .62]), competence (*individualist: k* = 20, *N* = 9881, ρ = .43 [CI .35, .51]; *collectivist: k* = 6, *N* = 1428, ρ = .33 [CI .20, .46]), and relatedness (*individualist: k* = 19, *N* = 10,003, ρ = .45 [CI .41, .49]; *collectivist: k* = 6, *N* = 1428, ρ = .53 [CI .40, .65]). We also had enough information to explore this moderator with respect to general well-being, distress, and the autonomous and controlled motivation variables.^3^ In each further comparison, there was no evidence of moderation.

Third, we explored publication status and how autonomy support was operationalized as potential study-related moderators. We considered publication bias using two approaches. Table 2 compares published versus unpublished studies (e.g., doctoral dissertations, conference presentations). For most variables, there was little evidence of publication bias, with only small differences in the estimated correlations between published and unpublished sources. In fact, in some cases the unpublished sources showed stronger, but very similar, correlations with LAS. However, LAS associations with indicators of eudaimonic well-being exhibited a clear stronger correlation in the published literature (*k* = 6, *N* = 2650, ρ = .47 [CI .35, .59]) than in the unpublished literature (*k* = 3, *N* = 990, ρ = .21 [CI .11, .31]). Given one subgroup in this analysis is based on only three studies, it is possible that this difference is an artifact of second order sampling error (Schmidt and Hunter 2015). Similar findings emerged for hedonic well-being and work performance, although there was some overlap in the CIs between the published and unpublished sources for these variables.

**Table 2:** Separated meta-analytic estimates for published and unpublished sources investigating leader autonomy support

Correlate	Published sources							Unpublished sources						
	*k*	*N*	*r*_*obs*_	ρ	*SD*_ρ_	95% CI		*k*	*N*	*r*_*obs*_	ρ	*SD*_ρ_	95% CI	
						LB	UB						LB	UB
Autonomous motivation	24	15,584	.34	.39	.07	.35	.42	7	1013	.30	.33	.12	.22	.44
Intrinsic motivation	15	12,507	.33	.39	.06	.35	.42	7	1147	.33	.36	.14	.19	.53
Identified regulation	9	8907	.26	.31	.05	.27	.34	3	769	.24	.29	.10	.15	.42
Controlled motivation	12	10,373	− .01	− .01	.18	− .11	.09	4	801	.17	.20	.09	.08	.31
Introjected regulation	9	8903	− .05	− .06	.20	− .19	.07	3	769	.14	.19	.00	.13	.25
External regulation	9	8909	− .02	− .02	.10	− .09	.06	3	769	.17	.19	.13	.03	.36
Autonomy orientation	7	3972	.20	.24	.12	.15	.37	6	469	.14	.16	.07	.04	.29
Basic needs total	23	11,234	.48	.55	.10	.50	.59	9	2109	.51	.58	.09	.51	.64
Autonomy need	15	8609	.45	.56	.08	.51	.60	10	2227	.50	.63	.13	.54	.72
Competence need	17	9122	.32	.39	.14	.32	.46	10	2514	.42	.51	.24	.36	.67
Relatedness need	17	9204	.36	.45	.07	.41	.49	9	2393	.43	.51	.12	.42	.59
General well-being	16	10,212	.41	.48	.12	.42	.54	10	2777	.34	.39	.17	.28	.50
Hedonic well-being	11	8223	.42	.50	.11	.43	.57	7	2119	.27	.32	.16	.19	.45
Eudaimonic well-being	6	2650	.40	.47	.14	.35	.59	3	990	.17	.21	.06	.11	.31
General distress	20	10,067	− .28	− .33	.09	− .37	− .28	5	1356	− .34	− .39	.15	− .53	− .24
Job satisfaction	13	6102	.49	.54	.11	.48	.61	9	1583	.52	.61	.15	.51	.71
Organisational commit	4	2241	.50	.55	.05	.49	.61	4	699	.39	.43	.00	.40	.47
Work engagement	11	4720	.28	.32	.05	.28	.36	7	1677	.33	.36	.06	.30	.43
Work performance	7	2039	.27	.31	.11	.22	.41	7	1220	.14	.16	.16	.03	.29
Prosocial behavior	9	3945	.23	.26	.10	.19	.33	4	870	.24	.26	.00	.22	.30

Meta-analytic estimates are only shown for variables which had *k* > 3 published and *k* > 3 unpublished sources. *k* number of studies in the analysis, *N* combined number of participants; *r*_*obs*_ sample size weighted mean observed correlation, ρ estimate of the true score correlation, *SD*_ρ_ standard deviation of estimated population correlation, *CI* confidence interval, *LB* lower bound, *UB* upper bound

We also used cumulative meta-analysis (CMA) to assess for bias as small studies were added into the meta-analysis (Schmidt and Hunter 2015). In CMA, studies are ranked on sample size and then added to the meta-analysis one at a time, starting with the study with the largest *N*. If publication bias is present, when the smaller sized studies are added it will cause the mean effect size to trend upwards (Borenstein et al. 2009; Schmidt and Hunter 2015). To increase the spread in the sizes of the studies for this procedure, we restricted these analyses to composite variables or those that had 20 studies or more. We compared meta-analytic estimates based on the largest *N* studies (only those above the median *N* in the set) against the full set with all smaller *N* studies included. If the 95% CIs across the two comparison meta-analytic estimates are mostly or entirely overlapping, then it suggests the results are not very different and bias is likely absent. Using this procedure, we found no evidence of small study bias across each variable, with each CI almost entirely or completely overlapping (see footnote 3). Overall, the lack of evidence for publication bias suggests that our findings are unlikely to be substantially biased in either a positive and negative direction and can therefore be interpreted with some confidence.

Finally, we explored whether correlations differed based on the autonomy support measure. Again, due to the variety of instruments used across studies and to establish an adequate number of studies per comparison, we limited these analyses to those variables containing 20 or more studies. We found little evidence of moderation, except for the LAS to general well-being association, where the Work Climate Scale to general well-being correlation was smaller (*k* = 3, *N* = 698, ρ = .11 [CI .01, .20]) than the 6-item Work Climate Questionnaire (*k* = 4, *N* = 1366, ρ = .42 [CI .31, .53]), and the 15-item Work Climate Questionnaire (*k* = 5, *N* = 1718, ρ = .46 [CI .37, .55]). Similarly, Perceived Autonomy Support Scale for Employees to general well-being correlation was higher (*k* = 4, *N* = 2012, ρ = .60 [CI .57, .64]) than the other measures. The absence of clear and consistent moderation effects across variables offers some support for the measures of LAS and suggests that findings based on any one measure are unlikely to be biased in either a positive or negative direction.

### Meta-analytic path analysis

The meta-analytically derived correlations matrix with associated samples is presented in Table 3. All correlations are corrected for sampling and measurement error. This matrix served as the input for the path analyses. Following the recommendations of Viswesvaran and Ones (1995), we calculated the squared multiple correlation for each variable, which is shown in the diagonal of the matrix. The harmonic mean of the sample sizes (*N* = 5238) was used as the input sample size for the analysis.

**Table 3:** Meta-analytically derived correlations and associated samples for variables in the path analysis

Variables		1	2	3	4	5	6	7	8	9
1	Leader autonomy support	.**51**	10,836 (25)	11,636 (27)	11,597 (26)	16,597 (31)	12,876 (26)	11,423 (25)	7782 (23)	6397 (18)
2	Autonomy need	.57	.**70**	45,824 (105)*	45,702 (104)*	12,253 (16)	5602 (16)*	10,369 (11)	1665 (8)	25,562 (50)*
3	Competence need	.42	.57*	.**61**	45,698 (104)*	12,438 (17)	5602 (16)*	10,431 (11)	1992 (9)	25,562 (50)*
4	Relatedness need	.46	.61*	.45*	.**65**	12,153 (15)	5602 (16)*	10,841 (12)	1554 (7)	25,971 (51)*
5	Autonomous motivation	.38	.48	.45	.36	.**50**	9638 (8)	12,856 (16)	6192 (14)	3820 (7)
6	General well-being	.46	.52*	.58*	.44*	.54	.**78**	10,015 (10)	2032 (8)	3318 (11)
7	General distress	− .33	− .61	− .64	− .64	− .35	− .75	.**85**	1538 (5)	3207 (8)
8	Positive work behavior	.27	.32	.40	.36	.42	.30	− .07	.**51**	7987 (15)
9	Work engagement	.33	.65*	.38*	.48*	.64	.62	− .47	.43	.**68**

Only retained variables in the model are shown; all correlations are corrected for sampling and measurement error; total *N* (*k*) used to calculate each estimate are shown above the diagonal; asterisk indicates data was taken from Van den Broeck et al. (2016); Squared Multiple Correlations are shown in bold in the diagonal; Harmonic mean of the sample sizes, *N* = 5667; autonomous motivation includes composites of identified regulation and intrinsic motivation; Positive work behavior includes composites of prosocial behavior, proactive behavior, and work performance

The path analysis was performed using the lavaan package (Rosseel 2012) of the open source R software (version 3.3.1), using Ordinary Least Squares (OLS) estimation. OLS assumptions are less restrictive than maximum likelihood (ML), which assumes multivariate normality across all variables in the model. OLS is also consistent with other studies using similar approaches (e.g., Colquitt et al. 2000), and is preferable to ML estimation when the data are in the form of correlations as opposed to covariances (Colquitt et al. 2000; Cudeck 1989; Podsakoff et al. 1996).

**Fig. 3:**
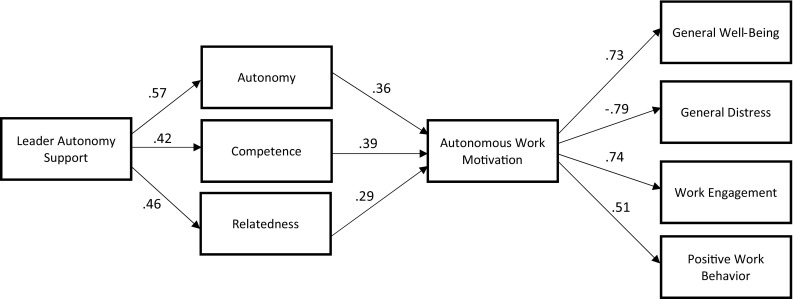
Path analysis diagram showing the patterns of relations among the variables in the study (*N* = 5667), with standardized parameter estimates. All paths are significant (*p* < .001); error variances are not shown for presentation simplicity. Chi square (*df* = 17) = 242.64, CFI = .977, TLI = .952, SRMR = .093, RMSEA = .048 [CI .043, .054]. Autonomous work motivation includes composites of identified and intrinsic motivation; positive work behavior includes composites of proactive behavior, prosocial behavior, and work performance

We first tested the full model shown in Fig. 2, allowing the variables in each block of the model to covary. However, results revealed evidence of multicollinearity with respect to some variables relating to both controlled work motivation and job satisfaction. On this basis, we removed these two variables from the analysis and re-ran the model using autonomous motivation as the sole mediator between basic needs and the remaining work outcomes. We calculated the new harmonic mean of the sample sizes (*N* = 5667), which was used as the input sample size. We again allowed the variables within each block of the model to covary. This revised model fit the data well (χ^2^ (17) = 242.644, CFI = .977, TLI = .952, RMSEA = .048 [CI .043, .054], and SRMR = .093). The final model is shown in Fig. 3 with standardized parameter estimates (for presentation simplicity, the intercorrelations and error variances are not shown in the diagram).

As can be observed in Fig. 3, LAS was most strongly associated with the need for autonomy, but also, as predicted, exhibited relatively strong associations with competence and relatedness needs. The three needs exhibited positive associations with autonomous work motivation, which in turn was strongly associated with the four health and work outcomes (general well-being, general distress, work engagement, and positive work behavior) in the predicted directions, after controlling for the autonomy, competence, and relatedness needs.

## Discussion

In this meta-analysis, we systematically combined and meta-analytically estimated the associations between perceived LAS and its correlates in the organizational literature, as well as moderators of those associations. Using meta-analytic path-analysis, we also tested an SDT derived model to confirm meditational processes consistent with SDT propositions in the workplace. Overall, our results are largely supportive of SDT predictions in organizations, suggesting that the provision of autonomy support may be a practical leadership approach to foster basic need satisfaction, the internalization of work motivation and positive work outcomes in individual employees. Below, we address the contribution and implications of the meta-analysis in more detail, and consider directions for future research.

### Study contributions and implications

First, our findings contribute to the literature by addressing previous inconsistencies in the strength of the reported relations between LAS and the motivational processes described by SDT. As we predicted (Hypothesis 1), our findings are consistent with the expected pattern outlined in Fig. 1, in that the provision of autonomy supportive practices was positively and progressively more strongly correlated with more internalized forms of motivation on the organismic integration continuum (Gagné et al. 2015; Ryan and Deci 2000). As such, LAS was most strongly associated with intrinsic motivation, was relatively unrelated to external regulation, and negatively related to amotivation. This finding is consistent with the premise that individuals are more likely to be autonomous and volitional in their work activities in an autonomy supportive context (Rigby and Ryan 2018).

Our findings also lend support to the overall pattern of correlations expected from the SDT literature (see Deci et al. 2017; Ryan and Deci 2000). LAS and employee basic needs for autonomy, competence, and relatedness were strongly and positively correlated. But supporting the findings of Van den Broeck et al. (2016) that each need predicts unique outcomes, LAS was most strongly associated with the need for autonomy. LAS yielded moderate to strong associations between employee well-being and positive work outcomes, suggesting that autonomy supportive approaches are consistent with thriving in the workplace.

The moderation analyses were surprisingly consistent; our results showed little evidence of moderation. Based on the transformational leadership literature, we expected that the source of autonomy support (proximal versus distal) would moderate correlations (Hypothesis 2), but results did not support this prediction. However, this result is consistent with a compensation effect of contextual factors on individual behavior and dispositions, which has been alluded to in prior work (e.g., Liu et al. 2011). That is, autonomy support from higher levels in the organization may substitute for more proximal autonomy support to trigger individual worker benefits. Alternatively, leader behaviors across different sources in the organizational hierarchies tend to be positively correlated (Liu and Fu 2011; Liu et al. 2011), which may attenuate source-related influences. As such, leaders often display similar behaviors to the ones they observe in others, potentially because they use such observations as a model for their own behavior (Brown et al. 2005). Still, few studies reported results separately for different sources of autonomy support, and future replications of our findings, exploring autonomy support at a variety of levels of social and physical distance in organizations, would be useful to determine whether or not autonomy support is indeed independent of its source.

Consistent with the universality hypothesis of SDT and consistent with our prediction (Hypothesis 3), we found no evidence of moderation effects as a function of whether studies were drawn from individualist versus collectivist countries. This finding lends some support to prior research showing that the relation between autonomy support and positive individual outcomes is robust beyond individualistic cultures (Chirkov et al. 2003, 2010; Ryan 1995) and is consistent with autonomy being a universal human need. Yet, while the present results are supportive of this SDT hypothesis, a note of caution is warranted. Although a sufficient number of studies in our analysis were conducted in countries that could be classified as individualist or collectivist using Hofstede’s (2001) classification, the samples within those studies were often comprised of participants with at least some mixed ethnic origins. This heterogeneity might hamper the detection of moderation. Thus, ongoing work should confirm this finding using diverse cultural groups.

An important theoretical contribution of the present study was achieved with the meta-analytic path analysis, which allowed us to go beyond the individual meta-analysis results by testing the theoretical ordering of SDT variables in the workplace specified by Deci et al. (2017). Results of these analyses supported SDT propositions (also confirming Hypotheses 4, 5, and 6), suggesting that LAS predicts well-being, engagement and positive work behavior through the mediational processes of basic need satisfaction and autonomous work motivation, respectively. Thus, our results confirm that LAS may be a critical social-contextual factor for fostering basic psychological needs and autonomous work motivation in employees.

Practically, our findings suggest that autonomy support may offer a valuable framework for leadership training interventions designed to engender thriving workforces. When done effectively, such interventions may help leaders to foster working environments that are conducive to employee basic need satisfaction and more volitional and autonomous work behavior, thus promoting increased mental health and well-being (Rigby and Ryan 2018). Indeed, preliminary experimental work on autonomy supportive training has suggested it can be effective in yielding positive outcomes for employees. For example, Hardré and Reeve (2009) found that an autonomy support training program increased autonomy supportiveness in management after 5 weeks, as well as corresponding levels of autonomous motivation and work engagement in their employees. This finding supported earlier intervention work (e.g., Deci et al. 1989), which showed autonomy support training to produce benefits in employee trust and job satisfaction. As both studies were based on small samples, we encourage further work of this nature so that effectiveness and return on investment from autonomy supportive training can be reliably established.

### Limitations and recommendations for future research

Despite the significant strengths of meta-analysis (Schmidt and Hunter 2015), it is important to acknowledge limitations of the present study. First, the studies included in the meta-analysis were mostly cross-sectional. It is possible that the relations in the proposed model are bi-directional. For example, autonomous motivation could engender higher levels of basic need satisfaction, and the presence of autonomous motivation might also prompt leaders to adopt a more autonomy supportive style (Ng et al. 2012). Nonetheless, our findings are consistent with the preliminary experimental work that indicates LAS is the antecedent (noted earlier), and we encourage further intervention studies with LAS as the predictor of other outcomes, as well as subsequent meta-analyses of intervention research to confirm LAS as a definitive cause of important motivational processes and well-being outcomes.

Second, we recognize that for a handful of variables in the present study, as well as some moderator subgroups, the number of available studies was small, which increases the likelihood of variance caused by second-order sampling error (Schmidt and Hunter 2015). The results of our moderation analyses that are based on imbalanced subgroups should be interpreted with some caution. We encourage further examination of these associations using larger samples and more balanced sub-group sizes.

Third, the present meta-analysis focused exclusively on LAS, which is only one part of the social context in organizations. More recent work in SDT has applied more holistic notions of need-supportive behaviors, including competence and relatedness support, both of which are thought to explain incremental variance in motivation, well-being, and performance (Deci et al. 2017; Ryan and Deci 2017). The other need supportive behaviors are strongly related with autonomy support (Deci et al. 2017), with studies often collapsing them into a single variable (e.g., Fernet et al. 2012), rather than establishing their unique contribution. Further research in the organizational context should explore their incremental predictive validity. In a similar way, future research needs to explore the effect of need-thwarting behaviors in organizations, such as controlling leadership (e.g., Trépanier et al. 2013), and how such behaviors affect well-being and performance. Currently, the predominantly studied variables in SDT are overwhelmingly positive behaviors (Van den Broeck et al. 2016), including LAS, and thus more needs to be known about the differential impact of need-thwarting behaviors and contexts (Bartholomew et al. 2011).

Finally, almost all of the measures in the studies were self-report instruments, which raises the possibility that our results are affected by self-report bias and shared method variance (Podsakoff et al. 2003). The one exception was work performance, which some studies measured objectively or with other-reports. Our findings did indicate clear evidence of moderation in the associations between LAS and work performance, whereby self-reported performance associations were higher, suggesting a possible upward bias in effect sizes when self-reports are used. Although this is consistent with other studies on work performance (Borman 1991; Donaldson and Grant-Vallone 2002), to lend weight to our findings, it is necessary for future research to confirm our associations using a variety of measures where possible, including objective employee data (e.g., absenteeism, turnover rates), valid biological indicators, as well as an exploration of autonomy support from multiple levels of analysis.

## Conclusion

Our meta-analytic review has demonstrated that perceived LAS is an important predictor of positive individual outcomes in employees, with correlations that are generally consistent with SDT propositions in organizations (e.g., Deci et al. 2017; Gagné et al. 2018). LAS was positively associated with desirable employee outcomes, including the internalization of work motivation, well-being, work engagement, positive job attitudes, and desired job behaviors, and was negatively related with employee distress and undesired job behaviors. These findings, as well as early experimental work, suggest that autonomy support may serve as an important underpinning for the development of management and leadership training interventions to promote enhanced employee functioning in organizations.

## Electronic supplementary material

Below is the link to the electronic supplementary material.

Supplementary material 1 (DOCX 71 KB)

## Footnotes

1 Scales were the Work Climate Questionnaire (WCQ, Baard et al. 2004), the Work Climate Scale (WCS, Deci et al. 1989), the Perceived Autonomy Support Scale for Employees (PASS-E, Mareau & Mageau, 2012), and the Personal and Institutional Autonomy Support scale (Lynch et al. 2005).

2 An insufficient number of studies were available for integrated regulation, likely because an integrated regulation subscale is often omitted from work motivation scales due to difficulty in statistically separating it from both the identified and intrinsic motivation subscales (See Gagné et al. 2015 for a discussion of this).

3 For space reasons, specific moderation results for the country of the study and the cumulative meta-analyses are not fully reported but are available by request from the first author.
