# Supplementary material for: Leader autonomy support in the workplace: A meta-analytic review
Source: Motiv Emot. 2018 May 17;42(5):706–24. doi: 10.1007/s11031-018-9698-y (PMC6133074; doi:10.1007/s11031-018-9698-y)
Supplement: Supplementary file 1 — Supplementary material 1 (DOCX 71 KB) [file 11031_2018_9698_MOESM1_ESM.docx]

**SUPPLEMENTAL FILES**

**APPENDIX A**

**Systematic Search Flow Diagram**

N = 4607 publications initially identified through database searches or had cited established measurement papers (e.g., Baard et al. 2004; Deci et al. 1989).

N = 720 publications searched through reference list snowballing.

N = 716 articles identified as relevant after duplicates removed. Selected for further screening.

N = 112 selected for data extraction after application of inclusion-exclusion criteria.

N = 72 sources included in quantitative synthesis, reflecting data from 83 unique samples and 32,870 participants.

N = 40 sources removed for failing to provide sufficient information.

N = 604 sources removed for failing to meet eligibility criteria.

**Year and citation**

**APPENDIX B**

**Meta-Analysis Coding Sheet**

Insert citation and year of study

**Sample**

*N* used to compute *r*

**Occupation of participants**

1. Insert occupation
2. Mixed
3. Not provided

**Country**

Insert country study conducted

**Published?**

1. Y
2. N

**IV measure**

Name and citation for Autonomy Support measure, and # items where necessary

**IV reliability**

Reliability coefficient for Autonomy Support measure

**Distance of Autonomy Support**

1. Proximal (immediate supervisor)
2. Distal (higher management)
3. Environment
4. Combination

**Global/specific DV**

1. Autonomous motivation (incl. intrinsic, integrated, or identified regulations)
2. Controlled motivation (incl. Introjected, external, or extrinsic regulations)
3. Amotivation
4. Work engagement
5. Intrinsic need satisfaction (incl. autonomy, competence, relatedness, or composite)
6. Intrinsic need thwarting
7. General motivation
8. Mindfulness
9. Well-being
10. Autonomy orientation
11. Control orientation
12. Psychological distress
13. Prosocial behavior
14. Proactive behavior
15. Job attitudes
    1. Job satisfaction
    2. Organizational commitment (affective, continuance, or normative, or composite)
16. Turnover intentions
17. Job performance (Objective, Self-report, Peer-report, Supervisor-report)
18. General health
19. Intention to stay
20. Absenteeism
21. Gender
22. Age
23. Tenure at organisation
24. Years’ experience in industry

**DV measure**

Name and citation for DV measure

**DV reliability**

Reliability coefficient for DV measure

***r***

Correlation coefficient between IV (AS measure) and DV

**Alternative to *r***

Insert data to estimate correlation between IV and DV if no correlation available

**Time lag**

Time between IV and DV measurement in months (if 0 write cross-sectional)

**APPENDIX C**

**Measured Variables Grouped Together to Construct Correlate Categories in the Meta-Analysis**

| **Correlate category** | **Measured variables** |
| --- | --- |
| **Autonomous motivation** | Autonomous motivation total  Intrinsic motivation  Identified regulation |
| **Controlled motivation** | Controlled motivation total  Introjected regulation  External regulation |
| **Basic needs total** | Basic needs total  Autonomy need  Competence need  Relatedness need |
| **General well-being** | Any measure of well-being, including constructs for hedonic and eudaimonic well-being, below:  Life satisfaction  Positive affect  Negative affect reverse  Hedonic well-being  General well-being  Self-esteem  Happiness  Subjective well-being |
| **Hedonic well-being** |  |
| **Eudaimonic well-being** | Personal accomplishment  Eudaimonic well-being  Psychological adjustment  Psychological empowerment |
| **General distress** | Somatization  General distress  Suicidal ideation  Negative affect  Depression  Health symptoms  Physical symptoms  The above, as well as measures for burnout and work stress, below:  Burnout total  Emotional exhaustion  Depersonalization  Personal accomplishment reverse  Cynicism  Inefficacy  Stress  Anxiety |
| **Burnout**  **Work stress** |  |
| **Organisational Commitment** | Organisational commitment total  Affective commitment  Normative commitment  Continuance commitment |
| **Job Satisfaction** | Job satisfaction  Career satisfaction |
| **Proactive Behavior** | Relationship building  Feedback seeking  Self-directed behavior  Voice  In-role behavior  Job crafting |
| **Prosocial Behavior** | Knowledge sharing  Helping behavior  Organizational citizenship behavior organizational  Organizational citizenship behavior individual |
| **Work Performance** | Self-report  Peer-report  Supervisor-report  Objective work performance |

*Note*: Correlate categories only shown for variables where heterogeneous construct measures were used to establish the correlate.

**SOURCES WITH STUDIES INCLUDED IN THE META-ANALYSIS**

Allen, J. B., & Bartle, M. (2014). Sport event volunteers’ engagement: management matters. *Managing Leisure*, *19(1)*, 36-50. <http://dx.doi.org/10.1080/13606719.2013.849504>

Arshadi, N. (2010). Basic need satisfaction, work motivation, and job performance in an industrial company in Iran. *Procedia Social and Behavioral Sciences*, *5*, 1267 - 1272. doi: 10.1016/j.sbspro.2010.07.274

Atkins, P. W. B., Hassed, C., & Fogliati, V. J. (2015). Mindfulness improves work engagement, wellbeing and performance in a university setting. In R. J. Burke, K. M. Page, & C. Cooper (Eds.), *Flourishing in life, work, and careers*. (pp. 193 - 209). Elgar: Cheltenham, UK.

Baard, P. P., Deci, E. L., & Ryan, R. M. (2004). Intrinsic Need Satisfaction: A Motivational Basis of Performance and Well-Being in Two Work Settings. *Journal of Applied Social Psychology*, *34*, 2045-2068.

Beenen, G., Pichler, S., & Levy, P. E. (in press). Self-determined feedback seeking: The role of perceived supervisor autonomy support. *Human Resource Management*. Doi: 10.1002/hrm.21789

Bélanger, J. J., Pierro, A., Barbieri, B., De Carlo, N. A., Falco, A., & Kruglanski, A. W. (2016). One size doesn’t fit all: the influence of supervisors’ power tactics and subordinates’ need for cognitive closure on burnout and stress. *European Journal of Work and Organizational Psychology*, *25(2)*, 287-300. doi: 10.1080/1359432X.2015.1061999

Beydogan (2008). Self-construal differences in perceived work situation and well-being. Unpublished doctoral dissertation.

Blais, M. R., & Brière, N. M. (2002). On the Mediational Role of Feelings of Self-Determination in the Workplace: Further Evidence and Generalization. Unpublished manuscript.

Braun, F. C., Avital, M., & Martz, B. (2012). Action-centered team leadership influences more than performance. *Team Performance Management*, *18*, 176-195. doi: 10.1108/13527591211241015

Chan, D. K-C., & Hagger, M. S. (2012). Autonomous forms of motivation underpinning injury prevention and rehabilitation among police officers: An application of the trans-contextual model. *Motivation and Emotion*, *36*, 349-364. doi: 10.1007/s11031-011-9247-4

Chang, Y., Leach, N., & Anderman, E. M. (2015). The role of perceived autonomy support in principals’ affective organizational commitment and job satisfaction. *Social Psychology of Education*, *18*, 315-336.

Choi, J-H. (2014). The effects of autonomous work environment and positive psychological capital on self-directed employee behavior: Evidence from Korea. Unpublished doctoral dissertation.

Cirka, C. C. (2000). Compliance and constructive contributions in organizations: Effects of managerial control styles on proactive employee behaviors. Unpublished doctoral dissertation.

Cirka, C. C. (2004). When actions speak as loudly as words: Autonomy support, psychological empowerment, and organizational citizenship behavior. In D. L. Turnipseed (Eds.), *Handbook of Organizational Citizenship Behavior* (pp. 289 - 325). Nova-Science Publishers.

Collie, R. J., & Martin, A. J. (2017). Teachers' sense of adaptability: Examining links with perceived autonomy support, teachers' psychological functioning, and students' numeracy achievement. *Learning and Individual Differences*, *55*, 29-39.

Collie, R. J., Shapka, J. D., Perry, N. E., & Martin, A. J. (2016). Teachers’ Psychological Functioning in the Workplace: Exploring the Roles of Contextual Beliefs, Need Satisfaction, and Personal Characteristics. *Journal of Educational Psychology*, *108(6)*, 788-799. <http://dx.doi.org/10.1037/edu0000088>

Dahling, J. J., & Lauricella, T. K. (in press). Linking Job Design to Subjective Career Success: A Test of Self-Determination Theory. *Journal of Career Assessment*.

Datu, J. A. D., & Mateo, N. J. (2016). Perceived Autonomy Support Moderates the Relations between Counseling Self-Efficacy and Flow among Filipino Counsellors. *Current Psychology*, *35*, 69-76. doi: 10.1007/s12144-015-9358-2

Deci, E. L., Ryan, R. M., Gagné, M., Leone, D. R., Usunov, J., & Kornazheva, B. P. (2001). Need Satisfaction, Motivation, and Well-Being in the Work Organizations of a Former Eastern Bloc Country: A Cross-Cultural Study of Self-Determination. *Personality and Social Psychology Bulletin*, *27(8)*, 930-942.

Delahaij, R., Theunissen, N. C. M., & Six, C. (2014). The influence of autonomy support on self-regulatory processes and attrition in the Royal Dutch Navy. *Learning and Individual Differences*, *30*, 177-181. http://dx.doi.org/10.1016/j.lindif.2013.11.003

Edgar, L. (1997). The Relationship Between the Characteristics of Nursing Care Delivery Systems and Work-Motivation, Satisfaction, and Intent to Leave. Unpublished doctoral dissertation.

Gagné, M. (2003). The Role of Autonomy Support and Autonomy Orientation in Prosocial Behavior Engagement. *Motivation and Emotion*, *27(3)*, 199-223. doi: 0146-7239/03/0900-0199/0

Gagné, M., Forest, J., Vansteenkiste, M., Crevier-Braud, L., Van den Broeck, A., Aspeli, A. K., Bellerose, J.,…& Westbye, C. (2015). The Multidimensional Work Motivation Scale: Validation evidence in seven languages and nine countries. *European Journal of Work and Organizational Psychology*, *24(2)*, 178-196. doi: 10.1080/1359432X.2013.877892

Ghorbani, N., & Watson, P. J. (2006). Validity of Experiential and Reflective Self-knowledge Scales: relationships with basic need satisfaction among Iranian factory workers. *Psychological Reports*, *98(3)*, 727-733.

Gillet, N., Fouquereau, E., Forest, J., Brunault, P., & Colombat, P. (2012). The impact of organizational factors on psychological needs and their relations with well-being. *Journal of Business and Psychology*, *27(4)*, 437-450.

Gillet, N., Colombat, P., Michinov, E., Pronost, A-M., & Fouquereau, E. (2013). Procedural justice, supervisor autonomy support, work satisfaction, organizational identification and job performance: the mediating role of need satisfaction and perceived organizational support. *Journal of Advanced Nursing*, *69(11)*, 2560-2571.

Gillett, N., Gagné, M., Sauvagère, S., & Fouquereau, E. (2013). The role of supervisor autonomy support, organizational support, and autonomous and controlled motivation in predicting employees' satisfaction and turnover intentions. *European Journal of Work and Organizational Psychology*, *22(4)*, 450-460. doi: 10.1080/1359432X.2012.665228

Golden, J. B. (2015). An Examination of the Volunteer Coordinator's Influence on Church Volunteers' Intent-to-Continue at the Largest Protestant Churches. Unpublished doctoral dissertation.

González, M. G. (2012). Self-Determination Theory and the Health Gradient in the Workplace: Exploring Psychological Pathways to Improving Health. Unpublished doctoral dissertation.

Grover, S. L., Teo, S. T. T., Pick, D., & Roche, D. (in press). Mindfulness as a personal resource to reduce work stress in the job demands‐resources model. *Stress & Health*.

Güntert, S. T. (2015). The impact of work design, autonomy support, and strategy on employee outcomes: A differentiated perspective on self-determination at work. *Motivation and Emotion*, *39*, 74-87. doi: 10.1007/s11031-014-9412-7

Haivas, S., Hofmans, J., & Pepermans, R. (2012). Self-Determination Theory as a Framework for Exploring the Impact of the Organizational Context on Volunteer Motivation: A Study of Romanian Volunteers. *Nonprofit and Voluntary Sector Quarterly*, *41(6)*, 1195-1214. doi: 10.1177/0899764011433041

Halvari, H., Vansteenkiste, M., Brørby, S., & Karlsen, H. P. (2013). Examining antecedents and outcomes of part-time working nurses’ motives to search and not to search for a full-time position. *Journal of Applied Social Psychology*, *43*, 1608-1623.

Harder, M. (2008). How do rewards and management styles influence the motivation to share knowledge. Unpublished working paper.

Hewett, R. (2014). Examining the relationship between workplace rewards and the quality of motivational experience: A Self-Determination Theory perspective. Unpublished doctoral dissertation.

Jolibois, J. L. (2014). A Self-Concept-Based Approach to Motivation to Lead. Unpublished doctoral dissertation.

Jones, J. E. (2002). Self-determination theory as a model for motivation in a training context. Unpublished doctoral dissertation.

Jungert, T., Koestner, R. F., Houlfort, N., & Schattke, K. (2013). Distinguishing source of autonomy support in relation to workers' motivation and self-efficacy. *The Journal of Social Psychology*, *153(6)*, 651-666.

Klassen, R M., Perry, N. E., Frenzel, A. C. (2012). Teachers’ Relatedness With Students: An Underemphasized Component of Teachers’ Basic Psychological Needs. *Journal of Educational Psychology*, *104(1)*, 150-165.

Kong, D. T., & Ho, V. T. (2016). A self-determination perspective of strengths use at work: Examining its determinant and performance implications. *The Journal of Positive Psychology*, *11(1)*, 15-25. <http://dx.doi.org/10.1080/17439760.2015.1004555>

Krieger, L. S., & Sheldon, K. M. (2015). What Makes Lawyers Happy?: A Data-Driven Prescription to Redefine Professional Success. *The George Washington Law Review*, *83*, 554-627.

Leone, D. R. (1995). The Relation of Work Climate, Higher Order Need Satisfaction, Need Salience, and Causality Orientations to Work Engagement, Psychological Adjustment, and Job Satisfaction. Unpublished doctoral dissertation.

Liu, D., & Fu, P-P. (2011). Motivating Protégés’ Personal Learning in Teams: A Multilevel Investigation of Autonomy Support and Autonomy Orientation. *Journal of Applied Psychology*, *96(6)*, 1195 - 1208. doi: 10.1037/a0024716

Liu, D., Zhang, S., Wang, L., & Lee, T. W. (2011). The Effects of Autonomy and Empowerment on Employee Turnover: Test of a Multilevel Model in Teams. *Journal of Applied Psychology*, *96(6)*, 1305-1316. doi: 10.1037/a0024518

Lynch, M. F., Plant, R. W., & Ryan, R. M. (2005). Psychological Needs and Threat to Safety: Implications for Staff and Patients in a Psychiatric Hospital for Youth. *Professional Psychology: Research and Practice*, *36(4)*, 415-425. doi: 10.1037/0735-7028.36.4.415

Moreau, E., & Mageau, G. A. (2012). The importance of perceived autonomy support for the psychological health and work satisfaction of health professionals: Not only supervisors count, colleagues too! *Motivation and Emotion*, *36*, 268-287. doi:10.1007/s11031-011-9250-9

Moreau, E. (2010). Article 2: Promoting the Psychological Health of Medical Residents: The Importance of Autonomy Support, Self-Concordance, and Self-Awareness. Part of unpublished doctoral dissertation.

Nie, Y., Chua, B. L., Yeung, A. S., Ryan, R. M., & Chan, W. Y. (2015). The importance of autonomy support and the mediating role of work motivation for well-being: Testing self-determination theory in a Chinese work organisation. *International Journal of Psychology*, *50(4)*, 245-255. doi: 10.1002/ijop.12110

Nutka, A., Haueis, M., Spitzer, M., & Hille, K. (2011). Designing Learning Environments In Assembly Lines Through Self-Determination. *Procedia - Social and Behavioral Sciences*, *29*, 752-757. doi: 10.1016/j.sbspro.2011.11.301

Olafsen, A. (2017). The implications of need-satisfying work climates on state mindfulness in a longitudinal analysis of work outcomes. *Motivation and Emotion*, *41*, 22 - 37. Doi: 10.1007/s11031-016-9592-4

Olafsen, A. H., Halvari, H., Forest, J., & Deci, E. L. (2015). Show them the money? The role of pay, managerial need support, and justice in a self-determination theory model of intrinsic work motivation. *Scandinavian Journal of Psychology*, *56(4)*, 447-457. doi: 10.1111/sjop.12211

Oldham, G. R., & Cummings, A. (1996). Employee Creativity: Personal and Contextual Factors at Work. *The Academy of Management Journal*, *39(3)*, 607-634.

Oostlander, J., Güntert, S. T., van Schie, S., & Wehner, T. (2014) Leadership and Volunteer Motivation: A Study Using Self-Determination Theory. *Nonprofit and Voluntary Sector Quarterly*, *43(5)*, 869-889. doi: 10.1177/0899764013485158

Oostlander, J., Güntert, S. T., & Wehner, T. (2014). Linking Autonomy-Supportive Leadership to Volunteer Satisfaction: A Self-Determination Theory Perspective. *Voluntas*, *25*, 1368-1387. doi: 10.1007/s11266-013-9395-0

Otis, N., & Pelletier, L. G. (2005). A motivational model of daily hassles, physical symptoms, and future work intentions among police officers. *Journal of Applied Social Psychology*, *35*, 2193-2214.

Palo, J., & Rothman, S. (2016). Work engagement in the mining industry in South Africa: The role of tasks and relationships. *Journal of Psychology in Africa*, *26(3)*, 221-229. <http://dx.doi.org/10.1080/14330237.2016.1185901>

Parfyonova, N. (2009). Employee motivation, performance, and well-being: The role of managerial support for autonomy, competence, and relatedness needs. Unpublished doctoral dissertation.

Ramirez, S. A. (2013). An Investigation of Perceived Organizational Support from a Self-Determination Theory Perspective. Unpublished masters dissertation.

Ranđelović, K. & Stojiljković, S. (2015). Work climate, basic psychological needs and burnout syndrome of primary school teachers and university professors. *Teme - Časopis za Društvene Nauke*, *3*, 823-844.

Roca, J. C., & Gagné, M. (2008). Understanding e-learning continuance intention in the workplace: A self-determination theory perspective. *Computers in Human Behavior*, *24*, 1585-1604. doi: 10.1016/j.chb.2007.06.001

Roche, M. & Haar, J. M. (2013). A metamodel approach towards self-determination theory: a study of New Zealand managers' organisational citizenship behaviours. *The International Journal of Human Resource Management*, *24(18)*, 3397-3417. doi: 10.1080/09585192.2013.770779

Schneeberger, N. A. (2006). Predicting Self-Determined Motivation and Organizational Citizenship Behaviors: A Multi-Level Model of Cascading Beliefs. Unpublished doctoral dissertation.

Sexton, J. (2013). The Application of Self-Determination Theory to Employee Motivation in Irish Workplaces. Unpublished dissertation.

Schultz, P. P., Ryan, R. M., Niemiec, C. P., Legate, N., & Williams, G. C. (2015). Mindfulness, work climate, and psychological need satisfaction in employee well-being. *Mindfulness*, *6*, 971-985. doi: 10.1007/s12671-014-0338-7

Senécal, C., Vallerand, R. J., & Guay, F. (2001). Antecedents and outcomes of work-family conflict: Toward a motivational model. *Personality and Social Psychology Bulletin*, *27(2)*, 176-186.

Slemp, G. R., Kern, M. L., & Vella-Brodrick, D. A. (2015). Workplace well-being: The role of job crafting and autonomy support. *Psychology of Well-Being: Theory, Research, and Practice*, *5(7)*, 1-17. doi: 10.1186/s13612-015-0034-y

Sullivan, G. S., Lonsdale, C., & Taylor, I. (2014). Burnout in High School Athletic Directors: A Self-Determination Perspective. *Journal of Applied Sport Psychology*, *26(3)*, 256-270. doi: 10.1080/10413200.2013.853328

Tripathi (2010). Autonomy, motivation, and work outcomes: A cultural perspective on Self-Determination Theory. Unpublished doctoral dissertation.

van Schie, S., Güntert, S. T., Oostlander, J., & Wehner, T. (2015). How the Organizational Context Impacts Volunteers: A Differentiated Perspective on Self-determined Motivation. *Voluntas*, *26*, 1570-1590. doi: 10.1007/s11266-014-9472-z

Walker, W. A. (2002). Motivation at work: A partial test of the Vallerand (1997) Hierarchical model of intrinsic and extrinsic motivation. Unpublished doctoral dissertation.

Wellman, D. A. (2008). Campus tour guide motivation: The role of intrinsic need satisfaction and autonomy support. Unpublished doctoral dissertation.

Williams, G. C., Halvari, H., Niemiec, C. P., Sørebø, Ø., Olafsen, A. H., & Westbye, C. (2014). Managerial support for basic psychological needs, somatic symptom burden and work-related correlates: A self-determination theory perspective. *Work & Stress*, *28(4)*, 404-419. doi: 10.1080/02678373.2014.971920
